# Supplementary material for: Variation in spatial dependencies across the cortical mantle discriminates the functional behaviour of primary and association cortex
Source: Nat Commun. 2023 Sep 13;14:5656. doi: 10.1038/s41467-023-41334-2 (PMC10499916; doi:10.1038/s41467-023-41334-2)
Supplement: Supplementary file 1 — Supplementary Figure [file 41467_2023_41334_MOESM1_ESM.pdf]

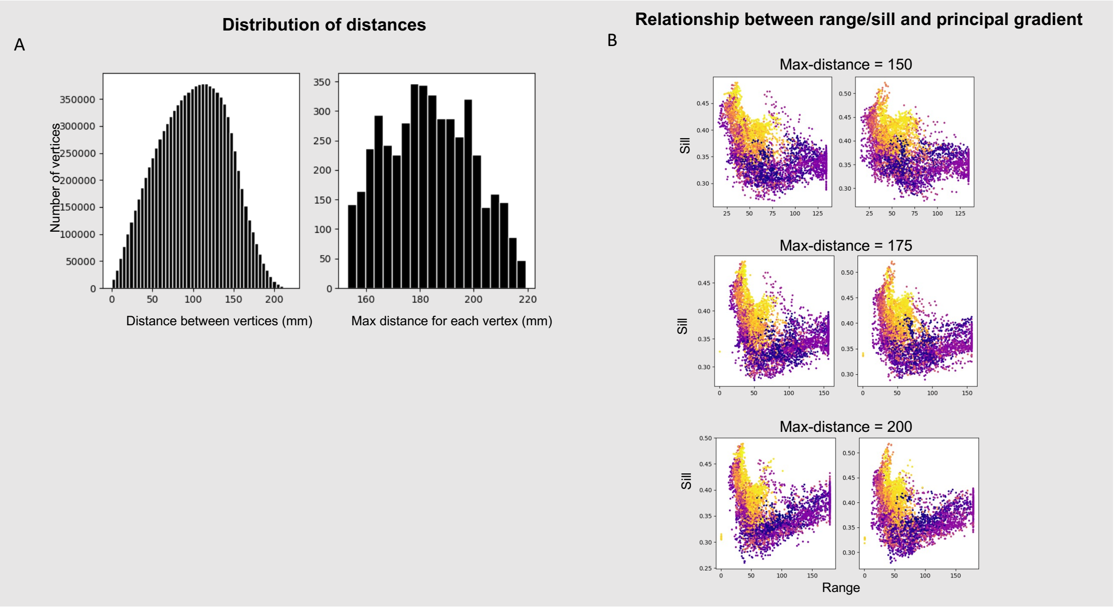

Supplementary Figure 1: A, distribution of geodesic distances (in mm) across all vertices and distribution of maximum distances for each vertex. B, the comparison of theoretical variogram parameters with the principal gradient, for different maximum distances when fitting the theoretical variograms.

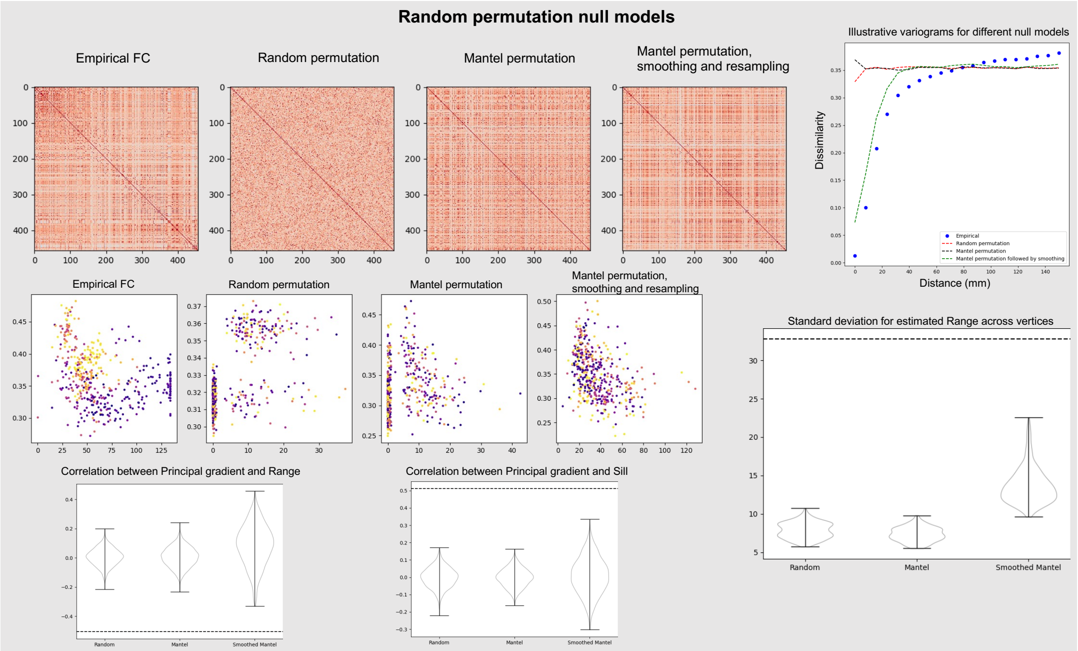

Supplementary Figure 2: Top, Connectivity matrices of empirical data, and three generative null models: random permutation, Mantel permutation, or Mantel permutation followed by homogeneous spatial smoothing and then resampling. Top right, the whole-brain variograms resulting from the different models. Middle: scatterplots displaying the relationship between principal gradient values and the sill and range of the theoretical variograms in the four different situations. Bottom, the distribution of correlation coefficients (between range and sill and the principal functional gradient) from generating 1000 iterations of each type of null model. The true, empirical value for the range and sill is depicted by the dashed lines and is outside the distribution for any of the null models. Bottom, right, the distribution of standard deviation of the range parameter (across vertices) for the 1000 generated models (true standard deviation is depicted by the dashed line). This illustrates the reduced heterogeneity of variograms across different vertices for the generated random models compared to the empirical data.
