# Supplementary material for: Variation in spatial dependencies across the cortical mantle discriminates the functional behaviour of primary and association cortex
Source: Nat Commun. 2023 Sep 13;14:5656. doi: 10.1038/s41467-023-41334-2 (PMC10499916; doi:10.1038/s41467-023-41334-2)
Supplement: Supplementary file 3 — Reporting Summary [file 41467_2023_41334_MOESM3_ESM.pdf]

nature portfolio

Corresponding author(s): Robert Leech

Last updated by author(s): 13-08-2023

Reporting Summary

Nature Portfolio wishes to improve the reproducibility of the work that we publish. This form provides structure for consistency and transparency in reporting. For further information on Nature Portfolio policies, see our Editorial Policies and the Editorial Policy Checklist.

Statistics

For all statistical analyses, confirm that the following items are present in the figure legend, table legend, main text, or Methods section.

n/a	Confirmed	
☐	☒	The exact sample size (n) for each experimental group/condition, given as a discrete number and unit of measurement
☐	☒	A statement on whether measurements were taken from distinct samples or whether the same sample was measured repeatedly
☐	☒	The statistical test(s) used AND whether they are one- or two-sided<br>*Only common tests should be described solely by name; describe more complex techniques in the Methods section.*
☒	☐	A description of all covariates tested
☐	☒	A description of any assumptions or corrections, such as tests of normality and adjustment for multiple comparisons
☐	☒	A full description of the statistical parameters including central tendency (e.g. means) or other basic estimates (e.g. regression coefficient) AND variation (e.g. standard deviation) or associated estimates of uncertainty (e.g. confidence intervals)
☐	☒	For null hypothesis testing, the test statistic (e.g. F, t, r) with confidence intervals, effect sizes, degrees of freedom and P value noted<br>*Give P values as exact values whenever suitable.*
☒	☐	For Bayesian analysis, information on the choice of priors and Markov chain Monte Carlo settings
☐	☒	For hierarchical and complex designs, identification of the appropriate level for tests and full reporting of outcomes
☐	☒	Estimates of effect sizes (e.g. Cohen's d, Pearson's r), indicating how they were calculated

Our web collection on statistics for biologists contains articles on many of the points above.

Software and code

Policy information about availability of computer code

Data collection	No data was collected for this study
Data analysis	Python 3.9, code available: https://github.com/ActiveNeuroImaging/BrainVariograms.git

For manuscripts utilizing custom algorithms or software that are central to the research but not yet described in published literature, software must be made available to editors and reviewers. We strongly encourage code deposition in a community repository (e.g. GitHub). See the Nature Portfolio guidelines for submitting code & software for further information.

Data

Policy information about availability of data

All manuscripts must include a data availability statement. This statement should provide the following information, where applicable:

- Accession codes, unique identifiers, or web links for publicly available datasets
- A description of any restrictions on data availability
- For clinical datasets or third party data, please ensure that the statement adheres to our policy

The data used in this study are available from the Human Connectome Project (https://www.humanconnectome.org/study/hcp-young-adult/document/extensively-processed-fmri-data-documentation), the PRIMatE Data and Resource Exchange (https://fcon_1000.projects.nitrc.org/indi/indiPRIME.html) and Neurosynth https://neurosynth.org/analyses/topics/).

Research involving human participants, their data, or biological material

Policy information about studies with human participants or human data. See also policy information about sex, gender (identity/presentation), and sexual orientation and race, ethnicity and racism.

Reporting on sex and gender	For the human participants, 34 of 51 reported their sex as female and 14 as male. No additional information on gender was available.<br><br>For the Macaque monkey dataset, 12 were male and 2 female.
Reporting on race, ethnicity, or other socially relevant groupings	This information was not available. See https://www.humanconnectome.org/study/hcp-young-adult/document/extensively-processed-fmri-data-documentation
Population characteristics	See https://www.humanconnectome.org/study/hcp-young-adult/document/extensively-processed-fmri-data-documentation
Recruitment	See https://www.humanconnectome.org/study/hcp-young-adult/document/extensively-processed-fmri-data-documentation
Ethics oversight	See https://www.humanconnectome.org/study/hcp-young-adult/document/extensively-processed-fmri-data-documentation

Note that full information on the approval of the study protocol must also be provided in the manuscript.

Field-specific reporting

Please select the one below that is the best fit for your research. If you are not sure, read the appropriate sections before making your selection.

☒ Life sciences ☐ Behavioural & social sciences ☐ Ecological, evolutionary & environmental sciences

For a reference copy of the document with all sections, see nature.com/documents/nr-reporting-summary-flat.pdf

Life sciences study design

All studies must disclose on these points even when the disclosure is negative.

Sample size	Sample size was not formally calculated. The manuscript contains the following explanation: "Since we were not focused on across-participant or within-participant variability, and for computational efficiency, we focused only on two scans from a subset (51) of the whole Human Connectome Project dataset."
Data exclusions	No data was excluded
Replication	We include various replication and test-retest reliability assessments in the manuscript
Randomization	This is not relevant given we were not investigating individual differences or group differences and were not collecting primary data.
Blinding	There are no group comparisons, so blinding is not relevant.

Reporting for specific materials, systems and methods

We require information from authors about some types of materials, experimental systems and methods used in many studies. Here, indicate whether each material, system or method listed is relevant to your study. If you are not sure if a list item applies to your research, read the appropriate section before selecting a response.

Materials & experimental systems

n/a	Involved in the study	
☒	☐	Antibodies
☒	☐	Eukaryotic cell lines
☒	☐	Palaeontology and archaeology
☒	☐	Animals and other organisms
☒	☐	Clinical data
☒	☐	Dual use research of concern
☒	☐	Plants

Methods

n/a	Involved in the study	
☒	☐	ChIP-seq
☒	☐	Flow cytometry
☐	☒	MRI-based neuroimaging

Magnetic resonance imaging

Experimental design

Design type	Resting state
Design specifications	Full details are available here: https://www.humanconnectome.org/study/hcp-young-adult
Behavioral performance measures	No behavioural data was considered.

Acquisition

Imaging type(s)	Functional
Field strength	3
Sequence & imaging parameters	Full details available: https://www.humanconnectome.org/study/hcp-young-adult
Area of acquisition	Cortex

Diffusion MRI ☐ Used ☒ Not used

Preprocessing

Preprocessing software	Details available https://www.humanconnectome.org/study/hcp-young-adult. We used the extensively preprocessed dataset
Normalization	See above
Normalization template	See above
Noise and artifact removal	See above
Volume censoring	See above

Statistical modeling & inference

Model type and settings	Standard models were not fit to the data
Effect(s) tested	Standard effects were not tested (see the manuscript)

Specify type of analysis: ☒ Whole brain ☐ ROI-based ☐ Both

Statistic type for inference (See Eklund et al. 2016)	Not relevant given the approach to modelling
Correction	FDR where appropriate.

Models & analysis

n/a	Involved in the study
☐	☒ Functional and/or effective connectivity
☐	☒ Graph analysis
☐	☒ Multivariate modeling or predictive analysis

Functional and/or effective connectivity	Pearson's correlation
Graph analysis	Variogram quantification and modelling
Multivariate modeling and predictive analysis	Diffusion embedding
